# The Combination of Chest Wall Perforator Flaps and Surgeon-Performed Breast Ultrasound: An Effective Synergy to Expand the Boundaries of Breast-Conserving Surgery

**DOI:** 10.1245/s10434-025-18281-x

**Published:** 2025-09-12

**Authors:** Massimo Ferrucci, Francesco Milardi, Daniele Passeri, Elena Miglioranza, Paola Del Bianco, Giacomo Montagna, Alberto Marchet

**Affiliations:** 1 Breast Surgery Unit, Veneto Institute of Oncology IOV, IRCSS - Istituto di Ricovero e Cura a Carattere Scientifico, Padova, Italy; 2 Department of Surgery, Oncology and Gastroenterology, University of Padova, Padova, Italy; 3 Department of Vascular Surgery, Vita-Salute University School of Medicine, San Raffaele Scientific Institute, Milan, Italy; 4 Clinical Research Unit, Veneto Institute of Oncology IOV, IRCCS-Istituto di Ricovero e Cura a Carattere Scientifico, Padova, Italy; 5 Breast Service, Department of Surgery, Memorial Sloan Kettering Cancer Center, New York, NY USA

**Keywords:** Breast cancer, Breast conserving surgery, Intraoperative ultrasound, Chest-wall perforator flaps, Partial breast reconstruction, Mastectomy rates

## Abstract

**Background:**

Intraoperative ultrasound-guided breast-conserving surgery (IOUS) combined with chest wall perforator flaps (CWPFs) is a promising approach to avoid mastectomy, especially for patients with high anticipated resection-to-breast volume ratios (ARR) who would otherwise be ineligible for breast conservation.

**Methods:**

This study prospectively analyzed surgical, oncologic, and cosmetic outcomes for consecutive patients with stages 0 to III breast cancer who underwent IOUS with CWPF-based partial breast reconstruction at a single institution between 2022 and 2024.

**Results:**

The study enrolled 73 female patients. The median age was 57 years, and the median tumor size was 32 mm, with 43.8% of lesions being multifocal/multicentric. The median ARR was 30.2%. The flap types included lateral intercostal artery perforator (LiCAP, 53.4%), anterior intercostal artery perforator (AICAP, 8.2%), medial intercostal artery perforator (MICAP, 19.2%), lateral thoracic artery perforator (LTAP, 16.4%), and thoracodorsal artery perforator (TDAP, 2.7%). The median flap volume was 90 cm^3^ (interquartile range [IQR], 47–140.5 cm), corresponding to 127% of the median specimens’ volume (71.1 cm^3^). The median operation time was 112 min. The 30-day global complication rate was 16.4%. No flap losses occurred. The positive margin rate was 9.6%, requiring re-excisions (5.5%) and mastectomies (4.1%). Adjuvant radiotherapy was administered to 95.9% of the patients, with no flap-related complications. During a median follow-up period of 14 months, only one distant recurrence was experienced, and no deaths occurred. Both patient- and surgeon-assessed evaluations demonstrated excellent cosmetic outcomes. Lower scores were associated with postoperative complications, re-excisions, and horizontal scars. None of the patients would have preferred mastectomy, and 89% underwent CWPF-based surgery to avoid it.

**Conclusions:**

The combination of IOUS and CWPFs yielded favorable surgical, cosmetic, and short-term oncologic outcome. This approach effectively and safely expands the indications for breast conservation, avoiding mastectomies, particularly for patients with small-to-medium breasts and an unfavorable ARR.

**Supplementary Information:**

The online version contains supplementary material available at 10.1245/s10434-025-18281-x.

Breast-conserving surgery (BCS) followed by radiotherapy is the standard of care for early-stage breast cancer (BC).^1,2^ However, patients with an unfavorable tumor-to-breast volume ratio and those with tumors in challenging anatomic locations are at higher risk for positive margins^3^ or poor aesthetic outcomes^4^. During the past three decades, refined oncoplastic techniques have extended the indications for BCS.^5^ Despite growing evidence supporting oncologic advantages of BCS over mastectomy, mastectomy rates remain high worldwide.^6,7^

Chest wall perforator flaps (CWPFs) represent an innovative volume replacement technique for partial breast reconstruction (PBR) that can avoid mastectomy for selected patients. These pedicled dermo-adipose flaps, harvested from extra-mammary thoracic wall regions and vascularized by chest wall perforator pedicles,^8^ enable wide, oncologically safe resections while achieving excellent cosmetic outcomes and high patient satisfaction.^9^ Unlike traditional myocutaneous flaps, CWPFs spare the underlying muscle, minimizing donor-site morbidity and promoting faster recovery, reduced postoperative pain, and preserved shoulder function.^10^

Surgery with CWPFs is particularly suitable for patients with small-to-medium size, mildly ptotic breasts with high tumor-to-breast volume ratios, avoiding mastectomy and implant-based reconstruction.^10^ Alternatively, they represent a valuable option for patients with large ptotic breasts who might otherwise be candidates for therapeutic reduction mammoplasty but refuse contralateral symmetrization.

Intraoperative ultrasound-guided surgery (IOUS)^11^ is the only technique providing real-time visualization of the tumor and resection margins, enabling precise excisions with lower positive margin rates.^12^ Surgeon-performed ultrasound plays a key role even in CWPF surgery.^13^ It allows precise preoperative planning, including a detailed anticipated resection volume assessment and an appropriate donor-site selection based on evaluation of local tissue availability. Additionally, Doppler ultrasound ensures accurate identification and monitoring of perforator pedicles before, during, and after surgery.^14^ The combination of CWPF surgery with ultrasound guidance represents a promising synergy for expanding the boundaries of BCS to patients who traditionally would have required mastectomy.

We investigated the feasibility of this combined approach in terms of surgical, short-term oncologic, and cosmetic outcomes, aiming to evaluate its safety and effectiveness in avoiding mastectomy for selected patients

## Methods

This prospective observational cohort study was conducted between January 2022 and September 2024 at the Veneto Institute of Oncology, Padova, Italy. The protocol was approved by the local Ethics Committee, and all the patients provided informed consent. The methodology adhered to the Strengthening the Reporting of Observational Studies in Epidemiology (STROBE) guidelines.^15^

### Inclusion and Exclusion Criteria

The study enrolled consecutive patients 18 years old or older with newly diagnosed stages 0 to III BC (according to the American Joint Committee on Cancer [AJCC] 8th-edition staging system^16^) who underwent oncoplastic volume replacement using CWPFs combined with IOUS. Patients treated with neoadjuvantchemotherapy (NACT) and those with multifocal/multicentric tumors or extensive ductal carcinoma *in situ* (DCIS) also were included. All the included patients had at least 6 months of follow-up evaluation from the time of surgery.

Patient selection was based on a comprehensive preoperative clinical evaluation including tumor location, breast ptosis, tumor-to-breast volume ratio, and the anatomic feasibility of CWPF harvest. Eligibility for CWPF-based reconstruction was determined by the operating surgeons through clinical examination and preoperative ultrasound aimed at evaluating both the adequacy of lateral chest wall perforators and the availability of sufficient soft tissue volume.

All eligible patients were thoroughly counseled on the available surgical options, including mastectomy with or without reconstruction, therapeutic reduction mammoplasty with contralateral symmetrization (when anatomically appropriate), and BCS with CWPF-based PBR. Surgery with CWPF was proposed as a primary oncoplastic strategy, not as a fallback option, within a structured shared decision-making process. The final surgical plan was individualized based on oncologic considerations, anatomic suitability, and patient preference. All the patients included in this cohort voluntarily opted for BCS with CWPF-based reconstruction.

The exclusion criteria ruled out stage IV metastatic disease, extremely unfavorable tumor-to-breast volume ratio precluding BCS despite CWPF, personal history of ipsilateral BC or prior whole-breast radiotherapy, insufficient donor tissue on the chest wall, and unfavorable anatomy or insufficient number of suitable perforator vessels due to small caliber, weak Doppler signal, or an unfavorable course.

### Preoperative Assessment, CWPF Choice, and Volume Calculations

All the patients underwent comprehensive preoperative imaging, including digital mammography and breast ultrasound, supplemented when indicated by magnetic resonance imaging (MRI) or contrast-enhanced mammography (CEM). Contrast-enhanced imaging was routinely performed in cases of multifocal/multicentric disease, lobular BC, or recommendation by breast radiologists.

Selection of CWPF for PBR was primarily guided by tumor location, and flaps were classified based on their vascular supply, as previously described.^8^ Thoracodorsal artery perforator (TDAP), lateral thoracic artery perforator (LTAP), and lateral intercostal artery perforator (LICAP) flaps were used for outer-quadrant tumors, whereas anterior intercostal artery perforator (AICAP) flaps were used for lower-quadrant tumors, and medial intercostal artery perforator (MICAP) flaps were used for inner-quadrant tumors. All volumetric measurements were performed by the operating surgeons based on clearly defined schemes and standardized formulas.

The predicted CWPF volume was calculated as a rhombic prism using the formula

$$V = d1*d2*h/2,$$

where *d1* and *d2* represent the perpendicular axes of the flap's skin projection, and *h* is the flap thickness, estimated via ultrasound as the distance from the skin to the underlying muscle. The optimal resection volume (ORV) was estimated as an ellipsoid using the formula

$$V = 4/3\pi *l1*l2*l3,$$

where *l1, l2,* and *l3* correspond to half of each tumor dimension plus an additional 1 cm. Breast volume was preoperatively calculated using mammographic diameters according to the Rostas’ method.^17^ The anticipated resection ratio (ARR) was defined as the ORV-to-breast volume ratio and expressed as a percentage. In cases of multifocal/multicentric tumors, the ORV reflected the total volume encompassing all tumor foci plus an additional 1 cm of surrounding healthy tissue in all directions. A schematic illustration of these concepts is provided in Fig. S1.

### The Three-Step Approach to Ultrasound-Guided CWPF Surgery

A GE Healthcare LOGIQ S8 ultrasound system (GE HealthCare Technologies, Inc., Chicago, IL, USA), equipped with both a linear array probe (ML6-15) and a dedicated intraoperative “hockey stick” probe (L8-18i), was used throughout all phases of the procedure. All surgeries were performed by breast-dedicated surgeons with proficiency in IOUS and a minimum experience of at least 10 independently performed CWPF procedures.

### Presurgical Evaluation

**Fig. 1:**
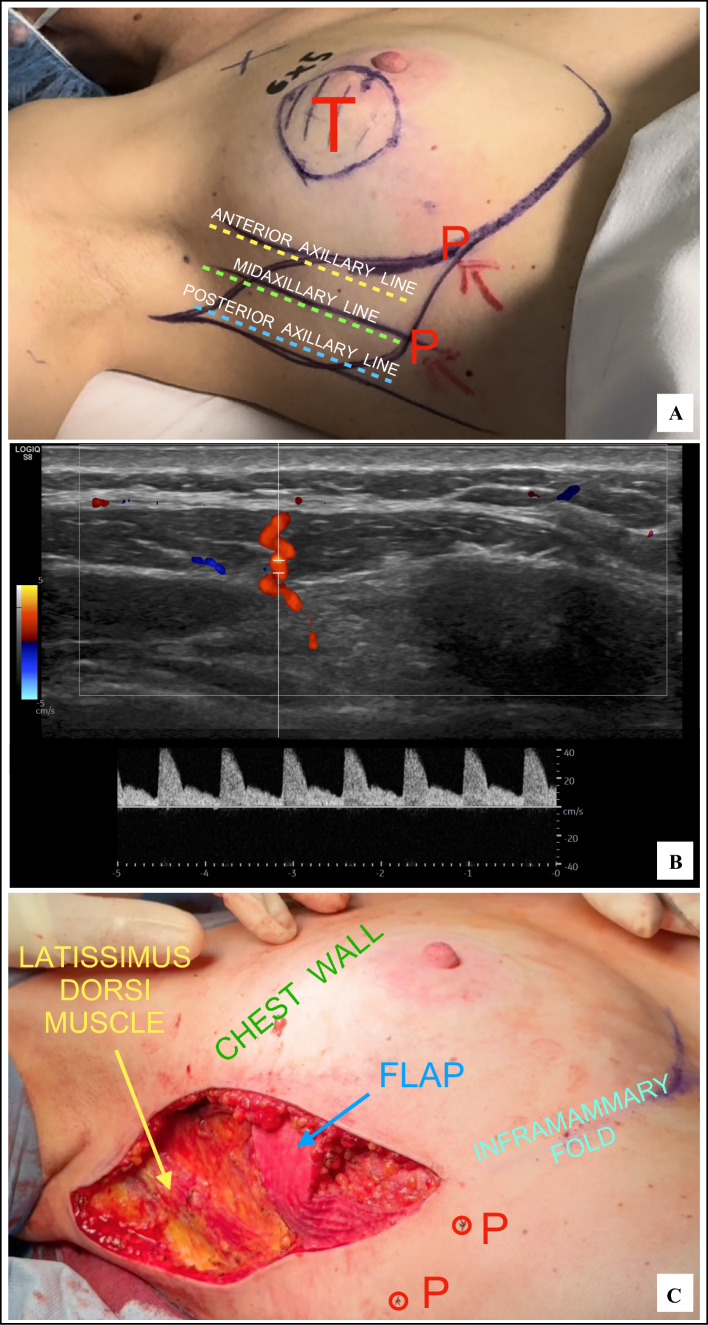
**A** Preoperative planning of an LICAP flap based on the vascular anatomy of chest wall perforators. **B** Power Doppler ultrasound image showing a LICAP pedicle with ideal peak systolic velocity. **C** Anatomic landmarks after flap movement in the final position. LICAP, lateral intercostal artery perforator; T, tumor; P, perforator

Based on tumor location, the potential CWPF donor sites then were examined, starting with a pinch test to evaluate soft tissue availability and to ensure primary skin closure without tension. A detailed ultrasound examination followed to confirm that the estimated flap volume would adequately replace the resection volume, targeting at least 110% of the ORV. Vascular anatomy was assessed using power-color Doppler (PCD) to verify the presence, caliber, and quality of perforators. Whenever feasible, dual afferent pedicles were sought to enhance vascular reliability. Finally, flap reach was assessed by simulating the arc of rotation with a flexible measuring tape to determine whether the anticipated flap length would allow for a tension-free transposition into the defect. The optimal flap mobilization technique—rotation (propeller or turnover) or advancement (pendulum-style movement)—also was determined. These comprehensive evaluations guided precise skin-marking and operative planning (Fig. 1A).

### Surgical Key Points

The IOUS-guided excisions were performed following a standardized technique.^11^

In cases of multifocal/multicentric tumors, a single en bloc resection including all tumor foci with adequate margins was performed to ensure oncologic safety and create a single reconstructive cavity. Intraoperatively, CWPF vascular pedicles were confirmed using PCD, with careful assessment of perforators and their peak systolic velocity (PSV). Pedicle suitability was evaluated following a well-established protocol^18–20^ involving transverse and longitudinal scanning with pulse-wave Doppler to confirm flow characteristics and exclude significant stenosis (Fig. 1B). Flap dissection was carefully Limited to within approximately 1 cm from the pedicles, preserving the loose perivascular tissue.

After complete flap elevation and reassessment of the optimal transfer strategy (turnover, propeller, or pendulum movement), the flap was moved to fill the resection defect and anchored to the residual gland using interrupted absorbable sutures (Fig. 1C). Local glandular reshaping was generally unnecessary. However, in selected cases with extensive defects, marked glandular laxity, or medially located tumors with limited flap length, minimal internal gland reshaping was performed before flap inset to optimize flap adaptation, enable tension-free suturing to the surrounding parenchyma, and enhance cosmetic contour. A gentle pressure maneuver applied to the skin overlying the flap, referred to as the “Ferrucci move,” was routinely performed to confirm the absence of surface depressions and to ensure a natural and effective volume replacement.

A final PCD check was performed with the flap in its definitive position to verify stable vascular flow. If altered Doppler waveforms were detected, flap repositioning was performed to relieve any tension or kinking until optimal vascular signals were restored. Quilting sutures^21^ were routinely applied to minimize the risk of seroma when permitted by the subcutaneous tissue.

### Follow-up Evaluations

Flap viability assessments via PCD were routinely performed before discharge, and subsequently during outpatient follow-up visits, or when clinically indicated. Postoperative recommendations were aligned with standard BCS protocols. All the patients were advised to wear a compression bra continuously for 1 month, with no particular restrictions on arm movement. However, the patients were instructed to avoid heavy lifting and strenuous physical activity during the first postoperative month.

All the patients underwent structured outpatient evaluations during the early postoperative period, depending on clinical need. Postoperative complications were systematically recorded and classified according to the Clavien-Dindo grading system.^22^ Scheduled follow-up evaluations were performed 4 weeks, 3 months, and 6 months postoperatively. Each clinical assessment included surgeon-performed breast ultrasound to assess pedicle pulsatility, evaluate flap thickness, monitor its changes over time (particularly after radiotherapy), and detect any fat necrosis or fluid collections.

Patient-reported satisfaction was evaluated at 6 months using an 11-item questionnaire adapted from the BREAST-Q Breast-Conserving Therapy Module,^23^ a validated tool for assessing cosmetic outcomes after BCS.^24^ Scores were converted through Rasch transformation according to the BREAST-Q protocol,^25^ resulting in a final score ranging from 0 to 100, with higher scores indicating greater satisfaction.

Aesthetic outcomes were independently evaluated by the surgical team through clinical evaluations performed 6 months after surgery, and at the most recent available follow-up visit. In addition, standardized pre- and postoperative photographs were taken for all the patients after their specific informed consent was received. Images were collected at four time points: before surgery and before discharge, then 1 and 6 months postoperatively. These photographs were used to complement clinical evaluation and support standardized cosmetic assessment across all follow-up visits. The three main authors (M.F., A.M., and F.M.) completed a standardized aesthetic assessment based on Aaronson’s criteria,^26^ evaluating breast shape and volume, contour deformity, nipple position, scar quality, skin changes, and overall appearance. These assessments were scored using the Harvard scale,^27^ a 4-point Likert-like system ranging from 1 (severe distortion) to 4 (nearly identical to the contralateral breast). In cases of disagreement, the mean of the three scores was used as the final rating.

### Statistical Analysis

Continuous variables are presented as medians and interquartile ranges. Comparisons between two groups were evaluated using the Mann-Whitney *U* test or two-sample *t* test, whereas comparisons across multiple groups were performed using the Kruskal–Wallis test. Categorical variables are presented as counts and percentages and were compared between groups using the chi-square or Fisher’s exact test, as appropriate. For assessing associations, uni- and multivariate Linear regression models were used, with corresponding 95% confidence intervals (CIs) and *p* values. The correlation between continuous variables was calculated using Spearman’s rank correlation coefficient.

All statistical tests used a two-sided 5% significance level, and a *p* value lower than 0.05 was considered statistically significant. Statistical analyses were performed using the RStudio software (RStudio: Integrated Development for R. RStudio, Inc., Boston, MA, USA).^28^

## Results

During the 33-month study period, 73 eligible Caucasian patients underwent IOUS-guided BCS and CWPF-based PBR. The median age was 57 years (interquartile range [IQR], 49–63 years), with 68.5% of the women post-menopausal at the time of surgery. All cases were unilateral, and 64.4% of the tumors presented as a palpable mass (Table 1). Contrast-enhanced imaging (MRI or CEM) was performed for 91.8% of the patients.

**Table 1:** Clinical characteristics and tumor features of the entire cohort

Clinical characteristics	Total (*n* = 73) *n/N* (%)	Tumor features	Total (*n* = 73) *n/N* (%)
Age (years)		Location	
Median (IQR)	57 (49–63)	Upper-outer quadrant	38 (52.1)
Range	36–76	Lower-inner quadrant	14 (19.2)
BMI (kg/m^2^)		Lower-outer quadrant	13 (17.8)
18–24.9	39 (53.4)	Upper-inner quadrant	8 (11.0)
25–29.9	29 (39.7)	Distribution	
30–34.9	4 (5.5)	Unifocal	41 (56.2)
≥ 35	1 (1.4)	Multifocal	23 (31.5)
Comorbidities		Multicentric	9 (12.3)
Cardiovascular disease	15 (20.5)	Palpable mass	
Diabetes mellitus	3 (4.1)	Yes	47 (64.4)
Smoking		No	26 (35.6)
No	68 (93.2)	Radiologic size (mm)	
Yes	5 (6.8)	Median (IQR)	32 (24–42)
Cup size		Range	8–94.4
A	14 (19.2)	Pathologic size (mm)	
B	38 (52.1)	Median (IQR)	29 (21–37)
C	16 (21.9)	Range	7–64.4
D	5 (6.8)	Histotype	
Ptosis		IC NST	39 (53.4)
Grade 1	48 (65.8)	ILC	21 (28.8)
Grade 2	23 (31.5)	DCIS	8 (11.0)
Grade 3	2 (2.8)	IC NST + DCIS	5 (6.8)
Fertility status		Biomolecular classification	
Pre-menopausal	23 (31.5)	Luminal A	34/65 (52.3)
Post-menopausal	50 (68.5)	Luminal B	19/65 (26.1)
Family history of breast cancer		Luminal B HER2-positive	3/65 (4.6)
Yes	28 (38.4)	HER2-positive	7/65 (10.7)
No	45 (61.6)	Triple-negative	4/65 (6.2)
Genetic test		Stage	
Performed, no mutation detected	6 (8.2)	0	11 (15.1)
Not performed	67 (91.8)	I	19 (26.0)
Treatment strategy		II	40 (54.8)
Neoadjuvant chemotherapy	14 (19.2)	III	3 (4.1)
Primary surgery	59 (80.8)		

IQR, interquartile range; BMI, body mass index; IC NST, invasive carcinoma of non-special type; ILC, invasive lobular carcinoma; DCIS, ductal carcinoma *in situ;* HER2, human epidermal growth factor receptor 2

Among the cohort, 53.4% had a body mass index (BMI) of 18 to 25 kg/m^2^. The most common cup size was B (52.1%), and the majority (65.8%) exhibited grade 1 ptosis according to the Regnault classification.^29^ Significant comorbidities were present in 24.6% of the patients. Five of the patients were active smokers, and 14 of the patients underwent NACT.

Most of the tumors (69.9%) were located in the outer quadrants. The median tumor diameter on preoperative imaging was 32 mm (IQR, 24–42 mm), and the median ORV was 65 cm^3^ (IQR, 42–102 cm^3^). With a median ARR of 30.2% (IQR, 18.6–41.7%; Table 2), and with 31.5% of the cases multifocal and 12.3% multicentric, 89% of the patients would have been candidates for mastectomy according to traditional surgical standards.

**Table 2:** Surgical and oncologic outcomes of the entire cohort

	Total (*n* = 73) *n/N* (%)		Total (*n* = 73) *n/N* (%)
CWPF type		PSV (cm/s)	
LICAP flap	39 (53.4)	Median (IQR)	15 (12–21)
MICAP flap	14 (19.2)	Range	8–29
Lateral thoracic intercostal artery perforator flap	12 (16.4)	Axillary surgery	
AICAP flap	6 (8.2)	SLNB	60 (82.2)
Thoraco-dorsal intercostal artery perforator flap	2 (2.7)	ALND	9 (12.3)
Indication to intervention		None	4 (5.5)
Avoid mastectomy	65 (89.0)	Drain	
Avoid bilateral reduction mammaplasty	8 (11.0)	Placed	7 (10.0)
Single-vessel based CWPF		Median LOS: days (IQR)	6 (4–12)
Yes	19 (26.0)	Hospital stay	
No	54 (74.0)	Day surgery	11 (15.1)
Incision site		Overnight stay	52 (71.2)
Lateral chest wall-vertical	34 (46.6)	> 24 h	10 (13.7)
Inframammary fold	20 (27.4)	Type of postoperative complications	
Lateral chest wall-horizontal	19 (26.0)	Total events	12 (16.4)
Skin paddle		Fat necrosis	6 (8.2)
Skin paddle included	4 (5.5)	Seroma	5 (6.8)
Fully de-epithelialized flap	69 (94.5)	Surgical-site infection	1 (1.4)
Movement of flap		Clavien–Dindo grade of complication	
Propeller	47 (64.4)	I	11/12 (91.7)
Pendulum	14 (19.2)	II	1/12 (8.3)
Turnover	12 (16.4)	Margins	
ARR (%)		Positive	7 (9.6)
Median (IQR)	30.2 (18.6–41.7)	Negative	66 (90.4)
Range	15.1–48.3	Margin management	
Flap volume (cm^3^)		Re-excision	4/7 (57.1)
Median (IQR)	90 (47–140.5)	Completion mastectomy	3/7 (42.9)
Range	30–300	Adjuvant therapies	
Specimen volume (cm^3^)		Radiation therapy	71 (95.9)
Median (IQR)	71.1 (46.1–114.2)	Endocrine therapy	54 (80.8)
Range	12.2–285.8	Chemotherapy	14 (19.2)
Operative time (min)		Trastuzumab	10 (13.7)
Median (IQR)	112 (84–135)	Recurrences	
Range	56.4–197.4	Locoregional recurrence	0 (0.0)
		Distant metastasis	1 (1.4)

CWPF, chest wall perforator flap; PSV, peak systolic velocity; LICAP, lateral intercostal artery perforator; IQR, interquartile range; MICAP, medial intercostal artery perforator; AICAP, anterior intercostal artery perforator flap; SLNB, sentinel lymph node biopsy; ALND, axillary lymph node dissection; LOS, length of stay; ARR, anticipated resection ratio

### Surgical Outcomes

Invasive carcinoma of no special type (NST) was the predominant histotype (60.3%), whereas invasive lobular carcinoma (ILC) accounted for 28.8% of all cases and was significantly associated with multifocal or multicentric distribution (*p* < 0.001). Luminal A was the most common subtype among all ICs (52.3%). Among the 13 patients with a preoperative diagnosis of extensive DCIS, 5 (38.5%) were upstaged to invasive carcinoma on final surgical pathology. Most tumors were classified as stage II (54.8%). The median pathologic tumor main diameter was 29 mm (IQR, 21–37 mm), and the median pathologic tumor volume was 8.6 cm^3^ (IQR, 3.5–18.4 8.6 cm^3^).

The median tumor-to-specimen volume ratio was 15.3% (IQR, 7.8–21.9%).

**Fig. 2:**
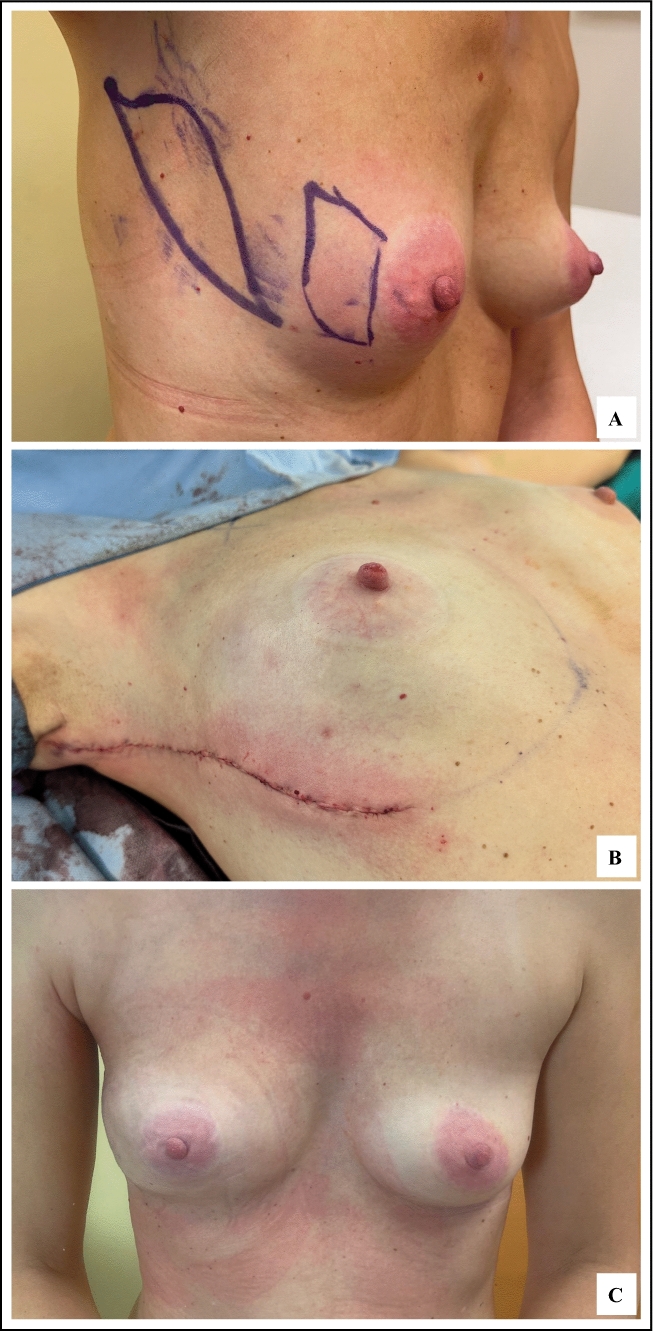
Partial breast reconstruction with an LICAP flap. **A** Preoperative planning of the resection area and estimated required flap volume. **B** Immediate postoperative appearance with scar placed outside the breast mound. **C** Postoperative day 10 image of a patient who underwent breast-conserving surgery for a pT2N0 right invasive breast cancer. LICAP, lateral intercostal artery perforator

The LICAP was the most frequently used flap (53.4%; Fig. 2). Only four patients required significant skin excision necessitating the use of a skin paddle. Of the 73 patients, 54 CWPFs were vascularized by a double pedicle, and 19 were based on a single pedicle. The median vascular pedicle PSV was 15 cm/s (IQR, 12–21 cm/s). The presence of a double pedicle was associated with larger flap volumes (median volume of 115.74 vs 73.53 cm^3^ in single-pedicle flaps; *p* = 0.002).

**Fig. 3:**
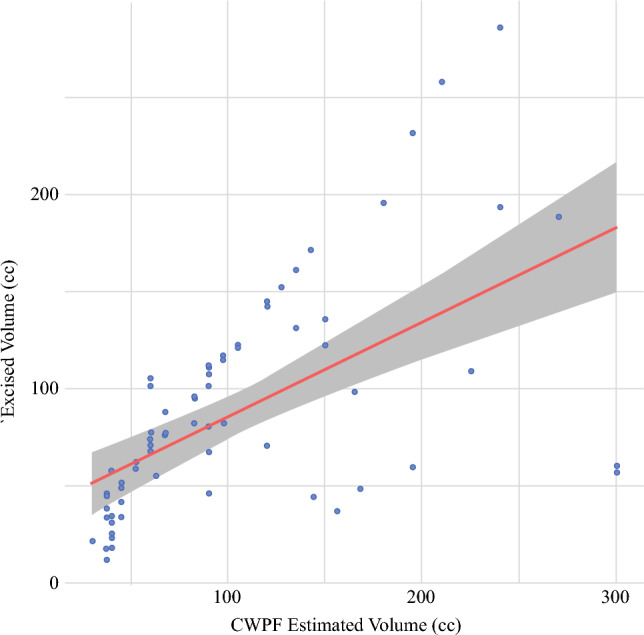
Scatter plot with linear regression analysis showing the correlation between excised volume and estimated chest wall perforator flap (CWPF) volume

**Fig. 4:**
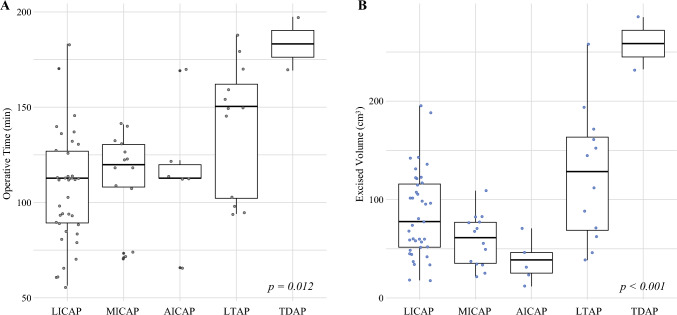
**A** Box plots illustrating the distribution of operative time. **B** Excised volume stratified by chest wall perforator flap type. LICAP, lateral intercostal artery perforator; MICAP, medial intercostal artery perforator; AICAP, anterior intercostal artery perforator; LTAP, lateral thoracic artery perforator; TDAP, thoraco-dorsal artery perforator

The median estimated flap volume was 90 cm^3^ (IQR, 47–140.5 cm^3^), corresponding to 127% of the median excised specimen volume, which was 71 cm^3^ (IQR, 46–110 cm^3^). A significant moderate-to-strong positive correlation was found between the CWPF preoperative estimated volume and the excised volume (*p* < 0.001; Fig. 3). The excised volumes across flap types differed significantly, with TDAP and LTAP flaps representing the largest median excised volumes (258.61 cm^3^ and 128.30 cm^3^, respectively; *p* < 0.001; Fig. 4).

The overall median operative time was 112 min (IQR, 84–135 min). The CWPF type significantly affected the median surgical duration (*p* = 0.012), with LTAP (150.4 min) and TDAP (183.3 min) requiring longer procedures. Axillary lymph node dissection (ALND) significantly prolonged operative time (131.6 vs 112.8 min without ALND; *p* = 0.011).

All the LTAP and TDAP flaps (19.2%) were mobilized in the final position with a pendulum movement. Among the other flaps, 64.4% were placed in the excision cavity with a propeller rotation and 16.4% using a turnover technique. Postoperative median vascular pedicle PSV was concordant with preoperative measurements (median, 14 cm/s; *p* = 0.740).

Surgical drains were selectively placed in only seven patients with elevated BMI or extensive excision volumes. The median time to drain removal was 6 days (range, 4–12 days). All surgical incisions were placed outside the breast, with the lateral chest wall as the most common incision site (72.6%).

As per routine practice at our institution, advanced oncoplastic procedures requiring general anesthesia typically involve overnight admission. Specifically, in case of extended CWPF-based PBRs, early postoperative monitoring of flap viability further supports this approach. Accordingly, the majority of patients (71.2%) required an overnight hospital stay, whereas 11 patients were safely managed as day-surgery cases.

The overall postoperative complication rate was 16.4%, with fat necrosis being the most frequent (8.2%; Table 2). All complications were managed conservatively in an outpatient setting, and only one case required antibiotic therapy. According to the Clavien-Dindo classification, 11 complications were grade I, and one was grade II. The median time to resolution was 15 days (range, 8–21 days). No cases of flap loss were observed. Notably, no chronic pain or upper limb functional impairments were reported. The patients who experienced seroma had significantly larger median excised volumes than those without seroma (231.46 vs 69.29 cm^3^, respectively; *p* < 0.001).

Among the patients with a single vascular pedicle supplying a large flap volume (>142.5 cm^3^, 75th percentile), the incidence of fat necrosis was significantly increased (*p* = 0.001; odds ratio [OR], 54.46). All cases of fat necrosis occurred in flaps with a PSV smaller than 15 cm/s (median, 11 cm/s), significantly smaller than in flaps without necrosis (*p* = 0.004), identifying this cutoff as a relevant risk factor.

Overall, the median ultrasound-assessed reduction in flap thickness at 6 months was 11.7%, decreased from a preoperative median of 20–18 mm. Larger PSV was significantly correlated with smaller 6-month flap thickness loss (*p* < 0.001). Consistently, 6-month flap thickness loss was significantly greater in the patients with fat necrosis than in those without fat necrosis (median, 22.1% vs 17.7%, respectively; *p* = 0.011), indicating an association between increased flap resorption and fat necrosis.

### Oncologic Outcomes

Positive margins were recorded in seven patients (9.6%) (4 with re-excision [all retaining the original flap for PBR] and 3 with completion mastectomy).

The risk of a positive margin was associated with the histologic subtype: three (37.5%) occurring in 8 patients with DCIS, four (16%) occurring in 25 patients with ILC (4/25), and nine occurring in 40 patients with IC NST (*p* < 0.001). This suggested that DCIS and lobular histology were associated with a higher risk of positive margins after initial surgery. Tumors with positive margins were significantly larger than those with negative margins (median volume, 16.69 vs 7.59 cm^3^; *p* = 0.048)

All the patients received adjuvant therapies (radiotherapy in 95.9%) without significant delay, with treatments typically initiated within 45 days after surgery, in accordance with institutional protocols and international recommendations. This likely reflects a streamlined institutional workflow, surgical team experience with CWPF-based reconstruction, and careful patient selection.

During a median follow-up period of 14 months (IQR, 9–16 months), one distant recurrence (bone) was recorded for a patient with triple-negative BC. No local recurrences were observed, and all the patients were alive at the time of the last follow-up visit.

### Cosmetic Outcomes

Cosmetic results were evaluated through both patient-reported outcomes and independent surgeon assessments. The BREAST-Q questionnaire, was completed by 67 patients (91.7% of the cohort). The global median Rasch-transformed score was 72 (IQR, 65–80), reflecting high patient satisfaction.

**Fig. 5:**
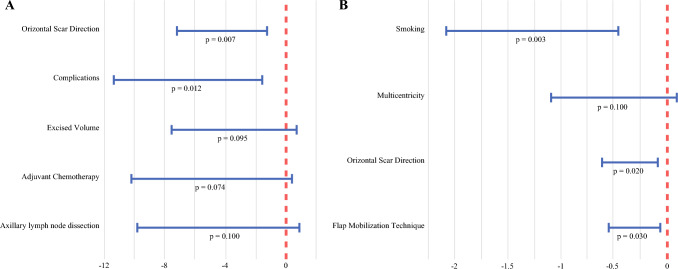
Multivariate forest plots illustrating the factors influencing **A** patients’ and **B** surgeons’ satisfaction with cosmetic outcomes

Univariate analysis identified significantly lower satisfaction scores for patients with larger excised specimen volumes (*p* = 0.0130), ALND (*p* = 0.005), postoperative complications (*p* = 0.031), need for re-excision (*p* = 0.041), and horizontal chest wall scars (*p* < 0.001). Multivariate analysis confirmed two independent predictors of reduced satisfaction: horizontal scar orientation (*p* = 0.007) and occurrence of postoperative complications (*p* = 0.012) (Fig. 5).

Importantly, a specific follow-up question assessing patients’ retrospective treatment preferences showed that none of the respondents would have opted for mastectomy over CWPF-based PBR, further highlighting the acceptability and satisfaction with this approach.

Surgeon-assessed cosmetic outcomes were consistent, with an overall median score of 3 (IQR, 2–4), indicating that in most cases, the treated breast appeared only slightly different from the breast on the contralateral side. Univariate analysis showed that worse scores were significantly associated with re-excisions (*p* = 0.043), multicentric tumor distribution (*p* = 0.052), horizontal scar orientation (*p* = 0.021), and smoking (*p* = 0.002). Conversely, pendulum and propeller flap mobilization techniques were associated with better outcomes (*p* = 0.034), likely due to an improved filling effect. Notably, the score was not significantly affected by tumor volume (*p* = 0.412) or type of CWPF used (*p* = 0.601).

Multivariate analysis identified smoking (*p* = 0.003), horizontal scar orientation (*p* = 0.020), and flap mobilization technique (*p* = 0.030) as independent predictors of poorer surgeon-assessed aesthetic outcomes.

## Discussion

This study confirmed that CWPFs for PBR represent a valuable alternative to mastectomy for selected patients, even in cases characterized by large excision volumes relative to breast size. Notably, our ARR was 30.2%, significantly higher than the typical threshold (>20%) associated with poorer cosmetic outcomes after standard BCS.^30^ The findings proved CWPFs to be reliable in reconstructing wide defects across all breast quadrants, and all skin incisions were positioned outside the breast mound, enhancing cosmetic results and preserving breast contour. The median operative time was 112 min, highlighting the feasibility of this approach, in line with previously reported series.^10^ Postoperative complications occurred in 16.4% of cases, comparable with the 10–25% complication rate reported for standard oncoplastic BCS or even lower.^31,32^ Importantly, no reoperation for aesthetic purposes or contralateral symmetrization was necessary, reflecting the volumetric precision and contouring capabilities of CWPFs.

### Safety and Efficacy of CWPFs

Since Hamdi et al.^8^ first introduced CWPFs for PBR in 2004, this technique has gained widespread interest as a reliable volume replacement option. Multiple studies have confirmed its oncologic safety and reproducibility.^33–35^

In a cohort of 150 patients, a 6.7% positive margin rate was observed, in Line with our findings, with an 86.4% 5-year disease-free rate of 86.4% and an overall survival rate of 94.7%.^33^ A large UK multicenter study involving patients with large or multicentric tumors showed acceptable complication (12%) and re-excision (15.9%) rates, reflecting the technique’s feasibility even in complex cases^34^.

Importantly, CWPFs have been shown to reduce mastectomy rates significantly. In a multicenter cohort of 603 women with multifocal or large-volume disease, two thirds underwent CWPF-based surgery specifically to avoid mastectomy (vs 89% in our study), with low complication (8.6%) and re-excision (15.9%) rates.^10^ During a median follow-up period of 22 months, the local and distant recurrence rates were 1.9% and 4.9%, respectively. Similar to our findings, most patients safely received adjuvant radiotherapy without flap-related issues.

Flap durability is a critical consideration. A recent study using MRI to assess flap volume over time showed that more than 80% of flap volume was retained 2 years after surgery in most patients.^36^ During a median follow-up period of 14 months, we observed no significant reduction in flap thickness over time even after radiotherapy, suggesting a stable volumetric and cosmetic outcome in the short-to-midterm.

### The Power of Surgeon-Performed Ultrasound: A Key Enabler for the Vertical Breast Surgeon

This study showed a novel and reproducible ultrasound-based method to evaluate flap vascular pedicle quality and preoperatively estimate the ARR and the required flap volume. This reinforces the central role of ultrasound, which must be performed directly by the breast surgeon as an essential tool for preoperative planning, intraoperative guidance, and postoperative monitoring of the procedure.

This multipurpose approach is supported by growing literature emphasizing the role of high-resolution PCD in flap planning, in which Doppler identification of perforators is critical.^13,14^ Handheld acoustic Doppler remains the most widely adopted tool for CWPF-based surgery due to its simplicity and accessibility. However, it provides only an approximate indication of pedicle location and pulsatility, with limited specificity and no visualization of anatomic structures.

In contrast, PCD ultrasonography offers a more advanced and informative alternative, enabling real-time visualization of vascular anatomy together with quantitative hemodynamic assessment. These features translate into superior sensitivity and accuracy, making PCD-ultrasound, in our view, an essential tool to optimize the performance and safety of CWPF-based PBR. In an LICAP-focused study, this flap was identified as an ideal like-for-like tissue for breast reconstruction, similar to the breast tissue for texture, color, and consistency. The authors describe PCD as the tool with the highest sensitivity and positive predictive value to identify perforating vessels, concluding that mastering ultrasound technology is the key to avoiding wrong flap design and eventual perforator damages.^13^

Tashiro et al.^14^ focused on the key role of PCD in LTAP flaps design, and described anatomic variants of this vessel. They listed the potential advantages of PCD: short procedural duration, low cost, absence of radiation exposure, ability to detect vessels in just the same surgical position, determination of their size and course, and the possibility of adapting the surgical plan depending on which LTAP branch is dominant.

Studies have already shown that IOUS is effective in locating and guiding the excision of all BC lesions.^11,12,37–42^ Despite its potential, IOUS still is underused, mainly due to an inexplicable lack of specific training,^43^ medicolegal concerns, or simple reluctance to embrace a new technique. In our experience, IOUS always guaranteed 100% localization and real-time visualization of the tumor and resection margins, allowing for precise tumor centralization within the specimen and low positive margin rates (9.6%) even in a similar population with very large excision volumes.

Taken together, these findings underscore the clinical relevance of integrating CWPFs and IOUS into modern BCS strategies. All the procedures were performed by breast surgeons with dedicated oncoplastic training, reinforcing the evolving role of breast surgeons as both oncologic and reconstructive breast specialists. Although CWPF-based PBR currently is limited to selected high-volume breast units and often performed by plastic surgeons, we advocate for broader adoption of this technique through the empowerment of breast surgeons with integrated excisional and reconstructive expertise.

### Cosmetic Impact of CWPF Surgery

This study demonstrated high levels of patient satisfaction after CWPF-based BCS, with no patients preferring mastectomy as an alternative. These findings align with limited existing data. An Italian series^44^ reported high satisfaction reported by approximately 80% of patients undergoing LTAP, LTD, and LICAP flap PBR, whereas van Zeelst et al.^45^ reported even higher scores than ours using the same BREAST-Q questionnaire (median score, 82 vs 72 for the breast satisfaction module, respectively). Notably, our study is the first to suggest that scar placement and flap movement direction may influence cosmetic outcomes, with horizontal scars and turnover movement associated with worse outcomes. Smoking was associated with worse cosmetic outcomes based on surgeons’ evaluations, highlighting the importance of patient selection and counseling.

Importantly, neither tumor volume nor flap type had a significant impact on satisfaction scores, reinforcing the feasibility of reconstructing all breast quadrants and confirming the reliable volume-replacement potential of CWPFs, even in the context of larger tumors.

### Study Strengths and Limitations

Our study had several strengths. It included diverse clinical scenarios, which increases the generalizability of its findings. Tumor, specimen, and flap volumes were calculated using standardized methods, ensuring consistency. To our knowledge, this is the first study to systematically evaluate the complete integration of IOUS with CWPFs in both the excisional and reconstructive phases of BCS, exploring a surgical approach entirely guided by surgeon-performed ultrasound.

The main limitations of the study were inherent to its non-randomized and unblinded design. The relatively short follow-up period limits the ability to draw conclusions on long-term oncologic outcomes. Although the use of a single BREAST-Q module ensured high compliance, it limited the breadth of the quality-of-life assessment and requires cautious interpretation of the aesthetic results. Despite a rigorous and standardized process for cosmetic evaluation, including direct clinical observation of all patients, the subjective nature of aesthetic assessment remains a limitation, further compounded by the absence of external reviewers and objective or technology-assisted evaluation tools.

Nevertheless, inter-observer agreement among the surgeon evaluators was high, and the concordance between patient-reported and surgeon-assessed outcomes reinforces the internal reliability of the results. The learning curve required to master this technique was not formally analyzed.

## Conclusions

This study demonstrated that the combination of IOUS and CWPF-based PBR is a safe, effective, and reproducible strategy to extend the indications for BCS, even for patients traditionally considered candidates for mastectomy, thus avoiding the need for implant-based reconstruction. This approach enables successful tumor excision in all breast quadrants while maintaining low complication rates, acceptable operative times, favorable short-term oncologic outcomes, and excellent cosmetic results.

The integration of surgeon-performed ultrasound throughout all surgical phases enhanced surgical precision and showed a strong synergy with CWPF-based reconstruction. Our findings support the expanding role of CWPFs in volume replacement, particularly in patients with small-to-moderate, non-ptotic or mildly ptotic breasts, as well as in those with larger breasts who decline therapeutic mammoplasty and contralateral symmetrization.

Beyond the encouraging clinical outcomes, this study also underscores the feasibility of integrating complex oncoplastic procedures, such as CWPF-based PBR, into routine breast surgical practice. The growing need to equip breast surgeons with both oncologic knowledge and reconstructive skills calls for structured training opportunities, including targeted oncoplastic fellowships, hands-on cadaveric courses, international exchange, and mentoring programs. We also advocate for the evolution of a modern “vertical” breast surgeon figure, capable of managing the entire continuum of care, from tumor excision to individualized reconstruction, in a unified, patient-centered approach. This integrated model not only improves surgical efficiency, but also offers a pragmatic response to workforce shortages, empowering breast surgeons to adopt advanced oncoplastic techniques through focused and dedicated training.

Overall, the efficacy and versatility of this combined technique offer a meaningful opportunity to reduce mastectomy rates. Nevertheless, larger studies with longer follow-up periods are needed to confirm these promising results and further define the long-term oncologic and aesthetic benefits of this integrated surgical approach.

## Supplementary Information

Below is the link to the electronic supplementary material.

Supplementary file1 (PDF 472 KB)

## Data Availability

All relevant data are within the paper and its supporting information files. Anything else required is available upon request.
