# Supplementary material for: The Combination of Chest Wall Perforator Flaps and Surgeon-Performed Breast Ultrasound: An Effective Synergy to Expand the Boundaries of Breast-Conserving Surgery
Source: Ann Surg Oncol. 2025 Sep 12;32(11):8538–51. doi: 10.1245/s10434-025-18281-x (PMC12494625; doi:10.1245/s10434-025-18281-x)
Supplement: Supplementary file 1 — Supplementary file1 (PDF 472 KB) [file 10434_2025_18281_MOESM1_ESM.pdf]

Chest Wall Perforator Flap volume

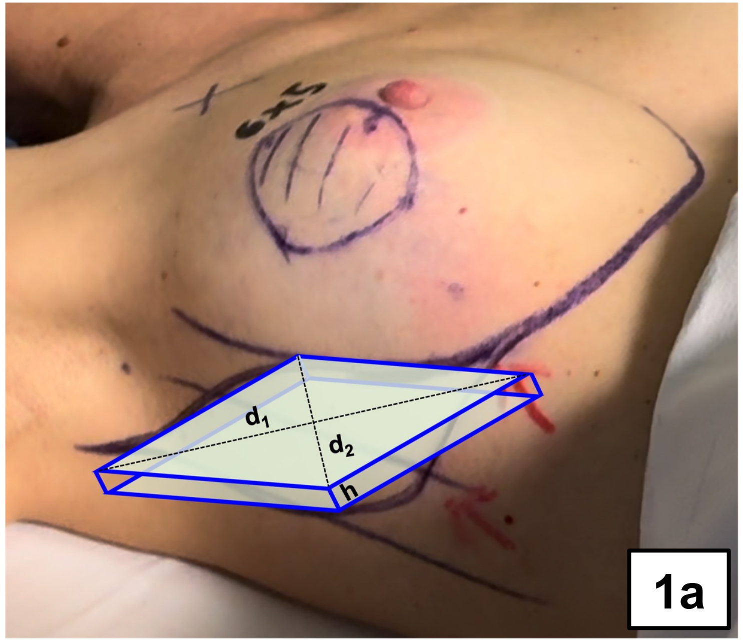

Tumor and Specimen volumes

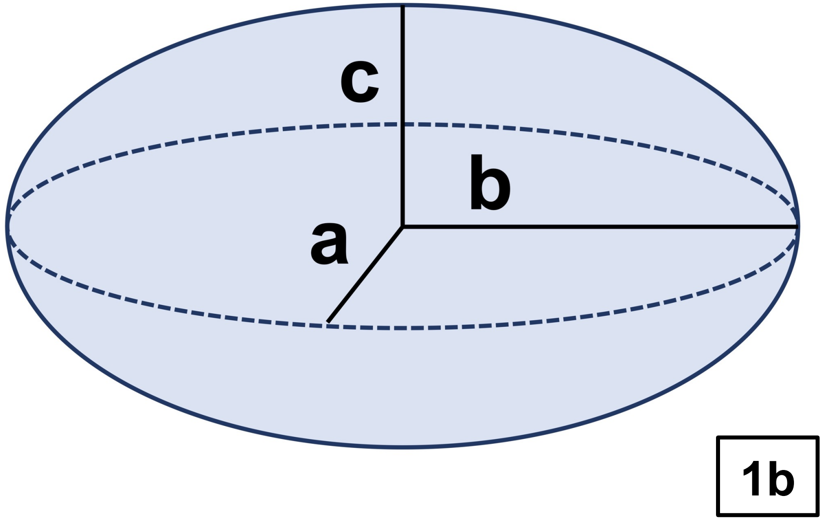

Optimal Resection Volume and Anticipated Resection Ratio

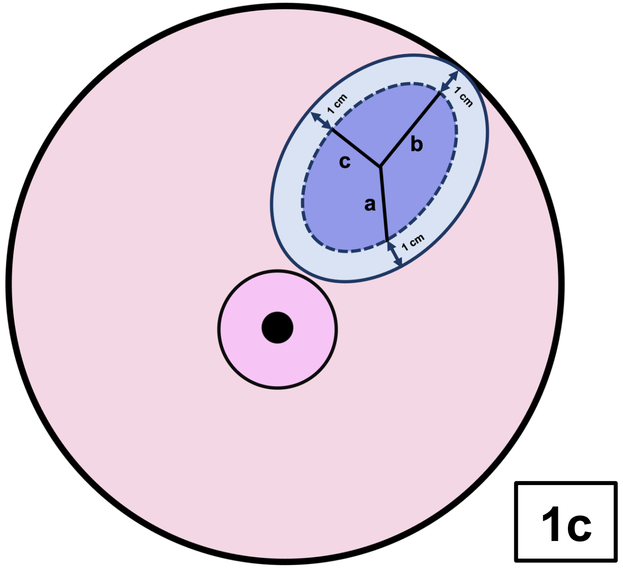

The predicted CWPF volume was calculated as a rhombic prism using the formula V= (d1*d2*h)/2, where d1 and d2 represent the perpendicular axes of the flap's skin projection, and h corresponds to the flap thickness, estimated via ultrasound as the distance from the skin surface to the underlying muscle fascia (**Supplementary Figure 1a**).

- Tumor and specimen volumes were calculated using the ellipsoid formula V=4/3*π*a*b*c, where a, b, and c represent the semi-diameters of the tumor (or the specimen) (**Supplementary Figure 1b**).
- The Optimal Resection Volume (ORV) was calculated by adding 1 cm margin in all directions, with en-block resections considered for multifocal/multicentric lesions (**Supplementary Figure 1c**).
- The Anticipated Resection Ratio was defined as ORV-to-breast volume ratio, and expressed as a percentage (**Supplementary Figure 1c**).

Supplementary Figure 1. Volumetric Assessment Workflow in Chest Wall Perforator Flaps based Partial Breast Reconstruction
